# Identification of the bHLH Factor Math6 as a Novel Component of the Embryonic Pancreas Transcriptional Network

**DOI:** 10.1371/journal.pone.0002430

**Published:** 2008-06-18

**Authors:** Francis C. Lynn, Lidia Sanchez, Ramon Gomis, Michael S. German, Rosa Gasa

**Affiliations:** 1 Diabetes Center, Hormone Research Institute, University of California San Francisco, San Francisco, California, United States of America; 2 Diabetes and Obesity Laboratory-Endocrinology and Nutrition Unit, Institut D'Investigacions Biomèdiques August Pi i Sunyer (IDIBAPS)-Hospital Clínic, Universitat de Barcelona, Barcelona, Spain; 3 CIBER de Diabetes y Enfermedades Metabólicas Asociadas (CIBERDEM), Barcelona, Spain; 4 Department of Medicine, University of California San Francisco, San Francisco, California, United States of America; University of Giessen Lung Center, Germany

## Abstract

**Background:**

Basic helix-loop-helix (bHLH) transcription factors play important roles in differentiation processes during embryonic development of vertebrates. In the pancreas, the *atonal*-related bHLH gene Neurogenin3 (Neurog3) controls endocrine cell fate specification in uncommitted progenitor cells. Therefore, it is likely that Neurog3-regulated factors will have important functions during pancreatic endocrine cell differentiation. The gene for the *atonal*-related bHLH factor Math6 was recognized as a potential target of Neurog3 in a genomic scale profiling during endocrine differentiation. Herein we have explored the role of Math6 during endocrine pancreas development.

**Results:**

We demonstrate that the *Math6* gene is a direct target of Neurog3 *in vitro* and that, during mouse development, Math6 is expressed in both endocrine and exocrine pancreatic precursor cells. We have investigated the role of Math6 in endocrine differentiation by over-expressing this factor in pancreatic duct cells. Math6 possesses intrinsic transcriptional repressor activity and, in contrast to Neurog3 it does not induce the endocrine differentiation program; however, it can modulate some of the pro-endocrine functions of Neurog3 in this system. In addition, we show that Math6 is broadly expressed in mouse embryonic tissues and its expression is induced by tissue-specific bHLH genes other than Neurog3. Furthermore, inactivation of the *Math6* gene in the mouse results in early embryonic lethality demonstrating an essential role of this factor in organismal development.

**Conclusions:**

These data demonstrate that Math6 is a novel component of the pancreatic transcriptional network during embryonic development and suggest a potential role for Math6 as a modulator of the differentiation program initiated by the pro-endocrine factor Neurog3. Furthermore, our results demonstrate that Math6 is indispensable for early embryonic development and indicate a more widespread function for this factor in tissue-specific differentiation processes that are dependent on class II bHLH genes.

## Introduction

During embryonic development, progenitor cells differentiate into the specialized cell types, which constitute the multicellular organism. Developmental transitions require rapid changes in gene expression as the progenitors progress through intermediate precursor states to differentiated cell types. In spite of the existence of lineage-specific differentiation programs, a few conserved types of molecular processes are often involved in the cellular mechanisms that control these transitions. One strategy that has evolved for this role is the activation of cascades of tissue-specific basic-helix-loop-helix (bHLH) transcription factors, which govern cell fate determination and differentiation in many tissues. A common theme in these programs is the transient expression of specific bHLH factors such as the neurogenins in brain and pancreas or myf5 in skeletal muscle that promote differentiation but whose expression is then repressed during differentiation to mature cells [1] [2] [3] [4] [5] [6]. Elucidating the cellular and molecular mechanisms that regulate initiation and termination of the expression of these differentiation factors will be important for understanding the developmental programs in these tissues.

The pancreas arises from dorsal and ventral aspects that bud from the pre-patterned gut endoderm at embryonic day (E)9 in the mouse. The epithelial cells within these buds expand and differentiate to generate the three major pancreatic lineages: endocrine islets of Langerhans, exocrine acini and pancreatic ducts. The endocrine cells that comprise the islets produce insulin (β), glucagon (α), somatostatin (δ), pancreatic polypeptide (PP) and ghrelin (ε). These endocrine cells are derived from progenitor cells that transiently express the basic helix-loop-helix factor Neurogenin3 (Neurog3). Loss-of-function experiments have demonstrated that Neurog3 is required for development of all endocrine cell lineages of the pancreas [7]. Conversely, gain-of-function approaches have shown that Neurog3 has the ability to drive the endocrine program [3] [8] [9] [10] [11] [12].

Neurog3 initiates the endocrine differentiation program but it is extinguished before final differentiation of the cells [2] [3]. The mechanism involved in disappearance of Neurog3 expression remains unclear. However, both Hes-1 [13] [14] and Neurog3 itself [15] are capable of repressing Neurog3 expression in endocrine progenitors and it has been proposed that induction by Neurog3 of a putative downstream repressor may participate in this negative autoregulatory loop [15].

A number of transcription factors participate in the process of differentiation and specification of islet cell subtypes downstream of Neurog3 including: the bHLH factor NeuroD1[16], the paired-box homeodomain factor Pax4 [17], the NK-homeodomain factors Nkx2-2 [18] and Nkx6-1[19], the homeodomain factor Arx [20] and the zinc finger-domain containing factor IA-1 [21]. Neurog3 is able to directly regulate the genes for NeuroD1, Pax4, Nkx2-2, and IA-1[22] [23] [24] [25] and these genes are likely to represent a minority of Neurog3 targets.

Despite major advances towards the identification of the molecular components of the islet cell lineage cascade [26] [27] [28], the full complement of factors necessary for endocrine development hasn't been defined. As a means of garnering further insight into potential factors involved in the developmental program, we have previously used adenovirally-transduced mouse pancreatic duct cells as a model for normal development. Following cDNA microarray expression profiling we identified a number of genes that are important during endocrine differentiation and function downstream of Neurog3 *in vivo* [9]. One of the genes mined using this approach was **m**ouse **at**onal **h**omolog **6** (Math6). As its name implies, Math6 is a member of the atonal superfamily of bHLH transcription factors that exhibits 43–57% identity in the bHLH domain with other mammalian atonal paralogs including the NeuroD and Neurogenin factors [29] [30]. Both Human (Hath6) and Drosophila (net) orthologs of this factor have complete sequence conservation within their bHLH domains and the Drosophila protein has been demonstrated to have important developmental roles [31] [32]. In the mouse, Math6 has been implicated in both neural and kidney development [29] [33]; however, its role in these programs remain unclear.

Here we have explored the role of Math6 in the pancreatic developmental program. We demonstrate that Math6 is expressed in the developing pancreas at the time when major differentiation of the endocrine and exocrine cell lineages occurs. Our data suggest that Math6 contributes to endocrine differentiation by modulating specific aspects of Neurog3 function. Additionally, we show that Math6 is crucial for early embryogenesis and propose that this factor participates in differentiation processes in other organs through specific interactions with tissue-specific bHLH proteins. Altogether, these data suggest roles for Math6 in both tissue-specific bHLH-dependent differentiation programs and in early embryonic development.

## Results

### *Math6* expression in the developing gut and pancreas

**Figure 1:**
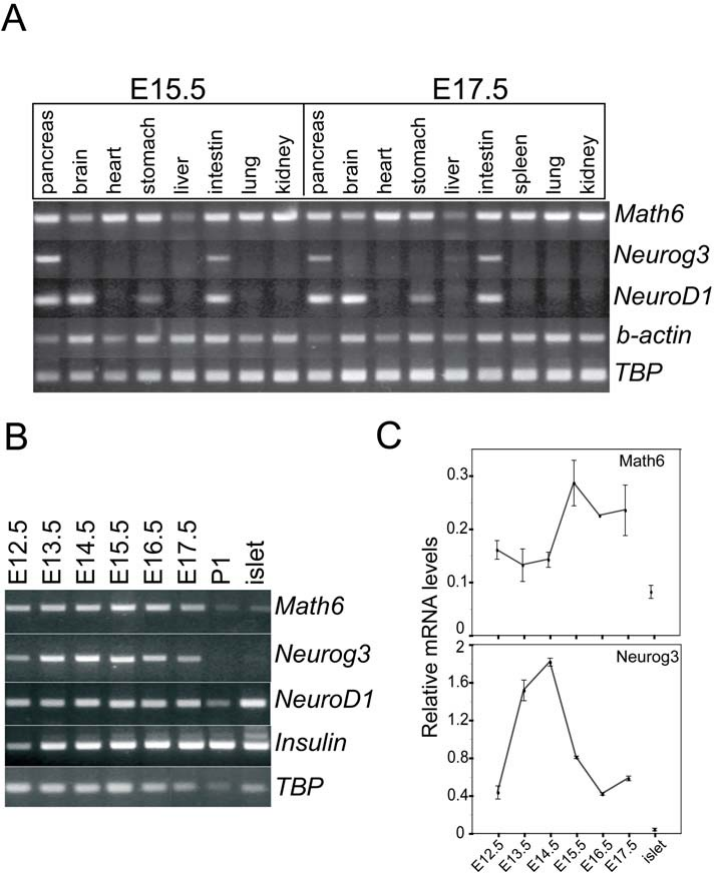
*Math6* mRNA is ubiquituosly expressed in mouse embryonic tissues. mRNAs encoding the atonal bHLH factors Math6, Neurog3 and NeuroD1 were assayed by RT-PCR of (A) mouse embryonic tissues at E15.5 and E17.5 and of (B) pancreatic tissue harvested at the indicated days of embryonic development, at postnatal day 1 (P1) and in adult pancreatic islets. Beta-actin (b-actin) and/or TATA-binding protein (TBP) were used as internal controls in RT-PCR assays (C) Relative levels of *Math6* and *Neurog3* mRNAs in pancreatic rudiments harvested at the indicated days of embryonic development were assessed by real time RT-PCR and expressed relative to GUS gene expression. Error bars represent standard error of the mean (SEM).

Prior studies in mice demonstrated Math6 expression in the developing brain, heart, kidney, lung and liver [29] [33], but did not test other organs. At embryonic days E15.5 and E17.5, we detected *Math6* mRNA in the pancreas, stomach, intestine and spleen as well (Figure 1A). Notably, *Math6* mRNA was expressed broadly in tissues derived from all three germ layers, unlike other related tissue-specific bHLH factors such as Neurog3 and NeuroD1 (Figure 1A).

Next, we determined the temporal expression profile of Math6 within the developing pancreas by RT-PCR (Figure 1B). We detected *Math6* mRNA in pancreatic tissue from E12.5 until E17.5 (Figure 1B). Math6 expression was still detectable at postnatal day 1 albeit at lower levels than in embryonic tissue, and a low level of mRNA expression remained in isolated pancreatic islets from adult mice (Figure 1B&C). The transient pancreatic expression profile of Math6 mirrors that of Neurog3 and differs from that of NeuroD1, which persists at very high levels in adult endocrine cells (Figure 1B).

Real time PCR was carried out to obtain quantitative estimates of the changes in expression levels of *Math6* and *Neurog3* during pancreatic development. As previously described, *Neurog3* mRNA expression increased 5-fold between E12.5 and E14.5 and then dropped to initial levels at E17.5. *Math6* mRNA levels changed 2-fold, reaching a peak at E15.5, one day later than that of *Neurog3* mRNA (Figure 1C). These results demonstrate that Math6 expression coincides with Neurog3 expression during embryonic pancreas development.

### Lineage-specific expression of Math6 in the pancreas

Next, we identified specific cell populations within the developing pancreas that express Math6. In the absence of suitable antibodies for immunohistochemical analyses, we used gene-targeting techniques to construct a mutant allele in which exons 1 and 2 of the Math6 gene were replaced with an enhanced Green Fluorescent Protein-Cre recombinase fusion protein (eGFP-Cre) (Figure S1). Mice heterozygous for the Math6 mutation (*Math6^+/EGFP-Cre^*) survived to adulthood and were indistinguishable from their wild type littermates.

**Figure 2:**
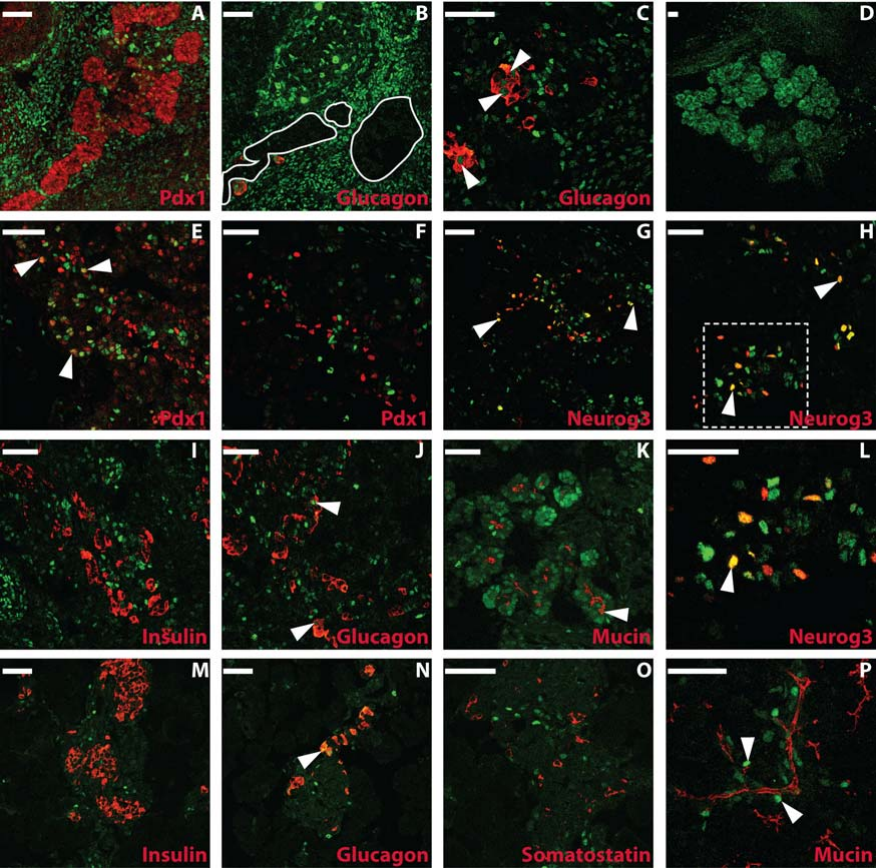
GFP expression in the mouse pancreas of math6 ^+/EGFP-Cre^ mice. Staining for GFP (Math6) in green is shown alone (D) or with Pdx-1(red; A,E,F), Neurog3 (red; G,H,L), glucagon (red; B,C,J,N), insulin (red; I,M), somatostatin (red; O) and mucin (red; K,P) at E12.5 (A–C), E14.5 (D) E15.5(E–L) and E 17.5(M–P). In B, pancreatic epithelium (absent of GFP staining) is outlined in white. Note that the epithelium coincides with the Pdx-1 expression domain as shown in panel A. In C, J and N, arrowheads indicate examples of cells coexpressing Math6 and glucagon. In E, arrowheads indicate cells coexpressing Math6 and Pdx-1. In G,H and L (H-Inset), arrowheads indicate cells coexpressing Math6 and Neurog3. In K and P, arrowheads indicate an example of cells coexpressing Math6 and mucin. Red and green channels are shown separately (scale bars = 50 µm).

We analyzed GFP immunofluorescence in *Math6^+/EGFP-Cre^* embryos as a surrogate marker of Math6 expression. GFP expression was not detected in the pancreatic buds at embryonic day E10.5 (data not shown). At E12.5, just prior to the peak of Neurog3 expression and endocrine cell differentiation, GFP+ cells were found in the developing pancreas. However, at this stage, GFP immunoreactivity appeared to be specifically excluded from the developing pancreatic epithelium, which is marked by Pdx-1 expression; although, staining was present in the early glucagon-positive cells and in the mesenchymal cells surrounding the nascent epithelium (Figure 2A–C).

By E15.5, GFP expression appeared in the pancreas in a subset of endocrine and exocrine progenitors (Figure 2E–L). The number of Neurog3+ progenitor cells peaks between E14.5 and E15.5, and we estimate that 50% of these Neurog3-immunoreactive cells expressed GFP at this time (Figure 2G,H&L). Math6 expression continued to be largely excluded from the Pdx-1 expression domain at E15.5 with the exception of some low-expressing Pdx-1 immunoreactive cells (Figure 2E&F). Notably, at E15.5, Neurog3+ cells are also specifically excluded from the Pdx-1 expression domain [3]. At this time point, robust Pdx-1 expression was restricted to fully differentiated β- and δ-cells and co-expression of GFP and insulin was not observed (Figure 2E&F). However, GFP immunoreactivity was observed in a subset of glucagon-positive cells (Figure 2J).

As observed with *Math6* mRNA expression (Figure 1), pancreatic GFP expression in *Math6^+/EGFP-Cre^* mice declined after E15.5 and reached undetectable levels in the adult organ. At E18.5, the remaining GFP-positive cells did not express insulin or somatostatin but did co-express glucagon and Mucin-1, a marker of ductal cells (Figure 2M–P and data not shown).

### Math6 is a direct gene target of Neurog3 *in vitro*

**Figure 3:**
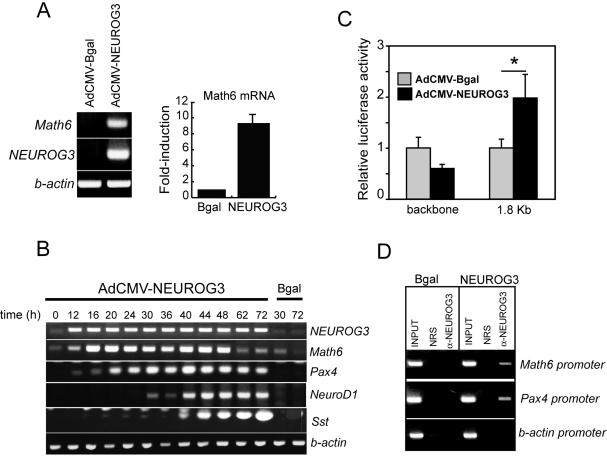
Math6 is a direct target of Neurog3. (A) *Math6* mRNA levels were increased in mPAC cells 48 h post-transduction with a recombinant adenovirus expressing Neurog3 (AdCMV.NEUROG3). Math6 mRNA expression was assessed by conventional RT-PCR (gel photograph of a representative RT-PCR experiment is shown) and quantitated by real time PCR using GUS as internal standard. Values are expressed as fold-increase over basal (AdCMV.Bgal-treated cells) and represent mean±SEM of 4 independent experiments. (B) Time course of induction of the indicated mRNAs in mPAC cells treated with AdCMV.NEUROG3 from time 0 to 2 h. This experiment is representative of 2 experiments with essentially identical results (C) AdCMV-NEUROG3 increased the transcriptional activity of a cotransfected reporter construct containing a 1.8 Kb *Math6* genomic fragment upstream of the luciferase gene. Luciferase activities are expressed relative to the activity of the reporter vector in Bgal-treated cells. Note that no increase in luciferase activity was observed with the backbone reporter vector. Bars represent mean±SEM for 3 experiments in triplicate. *p<0.05 vs. Bgal-treated cells (Student's t-test) (D) Cross-linked chromatin from mPAC cells treated with AdCMV.Bgal or AdCMV-NEUROG3 was immunoprecipitated with normal rabbit serum or an antiserum against human Neurog3. Immunoprecipitated DNA was analyzed by PCR with specific primers to the *Math6, pax4* and *beta actin* promoter regions. INPUTs correspond to 5% of total chromatin used per immunoprecipitation.

We had previously shown that Neurog3 activated Math6 expression in mPAC pancreatic duct cells [34], and we confirmed our previous microarray results using conventional RT-PCR and real time PCR (Figure 3A). *Math6* mRNA was expressed at low levels in untreated mPAC cells and was induced 12 h after infection with AdCMV-NEUROG3, concomitantly with appearance of *Pax4* mRNA and prior to induction of *NeuroD1* mRNA, two known direct targets of Neurog3 (Figure 3B). Interestingly, *Math6* mRNA expression declined 48 h post-infection, whereas levels of other Neurog3-induced mRNAs were maintained (*Pax4, NeuroD1*) or remained elevated (*somatostatin*) through 72 h. The observed decline in *Math6* mRNA expression did not result from any decrease in NEUROG3 transgene expression (Figure 3B). Importantly, the ability of Neurog3 to regulate *Math6* gene expression is conserved across species, as indicated by induction of *Hath6* mRNA in response to ectopic Neurog3 in the human pancreatic ductal cell lines PANC-1 and HPDE E6E7 (Figure S2).

The early induction of *Math6* mRNA by Neurog3 suggests that the Math6 gene may be a direct gene target of Neurog3. To determine if Neurog3 was able to directly activate *Math6* gene expression, we cloned a 1.8 Kb genomic fragment upstream of the mouse *Math6* translational start site and used it to drive expression of luciferase in pancreatic duct cells. Figure 3C demonstrates that, 24 hours after adenovirus-assisted transfection, Math6 promoter activity was approximately 2.0-fold higher in cells that had been transduced with Neurog3; indicating that Math6 transcription is regulated by Neurog3. Binding of Neurog3 to the Math6 promoter was further confirmed by chromatin immunoprecipitation (Figure 3D).

### Overexpressed Math6 represses Neurog3-induced gene activation events

**Figure 4:**
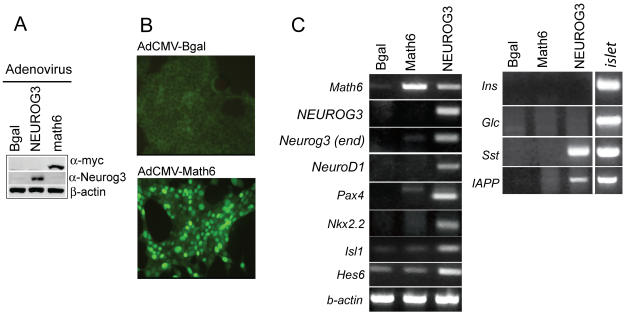
Overexpressed Math6 does not induce ectopic endocrine gene expression. mPAC cells were treated with equal doses of AdCMV.Bgal, AdCMV.NEUROG3 and AdCMV.Math6 and collected 48 h later for protein and RNA analysis (A) Immunoblot showing adenovirally-expressed Math6 (α-myc) and Neurog3 in total protein extracts (B) Immunocytochemistry showing nuclear localization of overexpressed Math6 (C) Representative RT-PCR showing mRNA levels for relevant endocrine transcription factors and endocrine cell genes. Experiments were repeated 3 times with identical results.

To investigate the function of Math6 in endocrine differentiation, we used an adenovirus encoding Math6 to express this factor in mPAC pancreatic duct cells. This treatment resulted in nuclear Math6 expression in these cells (Figure 4A–B). Math6 did not activate expression of the pancreatic transcription factors Neurog3, NeuroD1, Pax4, Nkx2.2, Nkx6-1, Isl-1, Pdx1, Hes6 and Hes1; nor did it induce the expression of the differentiated islet cell markers insulin, glucagon, somatostatin, PP, IAPP and glucokinase (Figure 4C and data not shown). Altogether, these results demonstrate that, unlike Neurog3 and Neurod1 transcription factors [9], [34], Math6 alone cannot induce differentiation or endocrine gene expression in this duct cell model.

**Figure 5:**
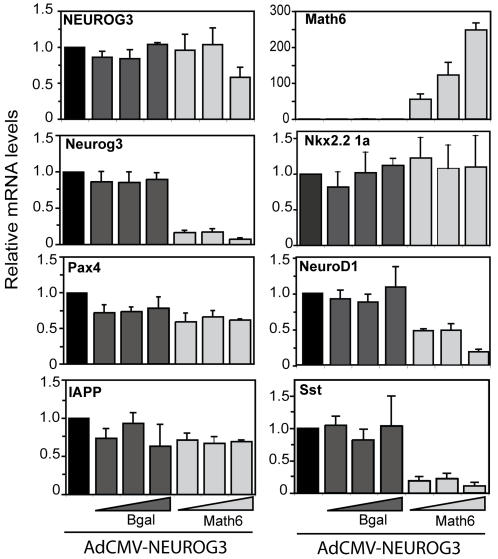
Overexpressed Math6 affects Neurog3 function in mPAC cells. mPAC cells were treated with AdCMV.NEUROG3 alone (m.o.i 40) or in combination with increasing doses (m.o.i. 20-40-100) of the adenoviruses AdCMV.Bgal and AdCMV.Math6. Total cellular RNA was isolated 48 h after viral treatment, and gene expression for the NEUROG3 transgene and the indicated endogenous genes were assessed by real time PCR and expressed relative to GUS gene expression. Bars represent mean±SEM for 3 to 4 independent experiments.

Since Math6 expression appears after Neurog3 *in vivo* and it is an early target of Neurog3 *in vitro*, we proposed that it might modulate Neurog3 function. We tested this hypothesis by comparing downstream target activation in cells transduced with AdCMV-NEUROG3 alone or in combination with increasing doses of AdCMV-Bgal or AdCMV-Math6 (Figure 5). We found that Math6 overexpression had no effect on Neurog3-induced activation of some genes (Pax4, IAPP, Nkx2-2) while it partially or totally inhibited the activation of others (Somatostatin, NeuroD1). Remarkably, Math6 significantly blocked Neurog3-induced activation of its own gene (Figure 5).

**Figure 6:**
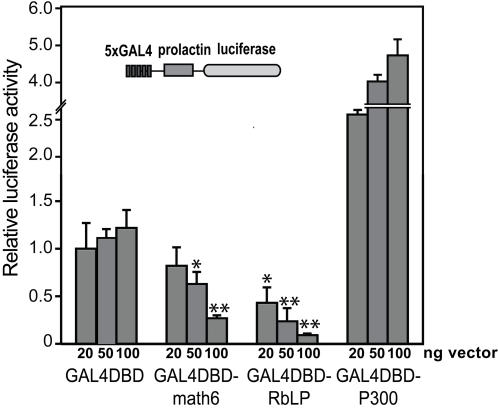
Math6 can function as a transcriptional repressor in vitro. A high background reporter plasmid consisting of five tandem copies of the Gal4 UAS upstream of the prolactin minimal promoter driving luciferase was cotransfected with increasing amounts of the plasmid expressing a fusion protein comprised of the GAL4 DNA binding domain (GAL4DBD) and full length Math6. Additionally, a GAL4DBD-Retinoblastoma Large Pocket (RbLP) and a GAL4-p300 (fragment 1737–2414 nt) fusion proteins were included as controls for repression and activation, respectively. Relative luciferase activities were calculated with the activity of cells transfected with the lowest amount of the GAL4DBD alone set at 1. All data are mean±SEM from transfections performed in duplicates on at least 4 occasions. * P<0.05. **P<0.01 as compared to equivalent amount of GAL4DBD vector (Student's t-test).

Our observations on the repressive function of Math6 on some Neurog3-targeting events is consistent with the activity of its Drosophila orthologue *net* which prevents wing vein formation by suppressing the function of vein-promoting genes [31]. To establish whether Math6 activates or represses transcription and if Math6 possesses an intrinsic repressive domain, we cotransfected an expression vector encoding full-length Math6 fused to a heterologous DNA-binding domain (GAL4-DBD) with a high basal activity reporter plasmid containing 5 copies of the GAL4 DNA binding site upstream of prolactin promoter-luciferase [35]. As shown in Figure 6, Math6 produces a nearly 5-fold transcriptional repression of this reporter construct in mPAC cells, implying that the Math6 protein can directly repress transcription.

**Figure 7:**
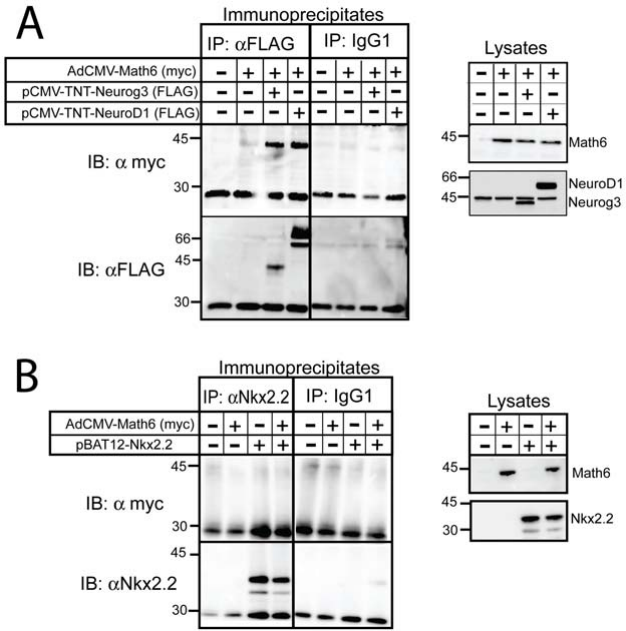
Math6 is immunoprecipitated with proendocrine bHLH factors. mPAC cells were treated with Ad-CMV-Math6 with or without expression vectors encoding Neurog3, NeuroD1 or Nkx2-2. 48 h after adeno-assisted transfection, cells were lysed and analyzed directly (lysates) or immunoprecipitated with α-FLAG, which recognizes the FLAG epitope tag at the N terminus of Neurog3 and NeuroD1, with α-Nkx.2.2 or with mouse IgG1. Precipitated proteins were resolved by SDS-PAGE and immunoblotted with α-myc, which recognizes the myc epitope tag at the N terminus of Math6, and with α-FLAG or α-Nkx2-2. Crude lysates equal 10% of the protein used in coimmunoprecipitations. Migration of MW markers (in KDa) are shown on the left.

To determine if the observed functional link between Math6 and Neurog3 resulted from a physical interaction between these two proteins, we carried out co-immunoprecipitation analyses. Using this approach, we were able to recover Math6 when Neurog3 was immunoprecipitated from the lysates of mPAC cells expressing both factors, thus demonstrating a physical association between Math6 and Neurog3 (Figure 7A). Next, we assessed whether Math6 was recovered with either the related bHLH factor NeuroD1 or the unrelated pancreatic homeodomain transcription factor Nkx2-2 and detected Math6 in NeuroD1 but not in Nkx2-2 immunoprecipitates (Figure 7A–B), implying that Math6 specifically associates with bHLH factors in this cellular context. While this interaction is not surprising since it is well established that bHLH proteins function as homo or heterodimers to regulate transcription, is should be noted that the association between Neurog3/NeuroD1 and Math6 could not be reproduced using *in vitro* translated proteins (data not shown). Thus it is plausible that the interaction between these factors must occur within the cell and/or involves accessory proteins and/or post-translational modifications.

### Math6 is necessary for initial stages of mouse embryonic development

We have demonstrated that Math6 is expressed in the developing pancreas and that it can modulate Neurog3 target gene expression *in vitro*. To determine its pancreatic role *in vivo*, we attempted to generate Math6 null mice by intercrossing *Math6^+/EGFP-Cre^* heterozygotes. We failed to obtain Math6 null animals among the progeny of such intercrossings, indicating that the Math6 mutant allele is likely embryonic lethal (82 pups; 55 *Math6^+/EGFP-Cre^*: 27 *Math6^+/+^*). In an attempt to determine when homozygous embryos were dying, embryos were isolated following timed matings as early as e8.5. From these crosses, approximately 1/4 implantations had grossly normal placental development but the embryo appeared to be developmentally arrested at or slightly after gastrulation. To assess whether germline presence of Math6 was important prior to gastrulation, blastocysts were isolated at e3.5 and genotyped. From these crosses knockout embryos were obtained (Figure S1-G) and GFP fluorescence was not observed. Unfortunately, early lethality of *Math6* null mice prevents the analysis of the function of this factor in pancreatic organogenesis; however, it does suggest that Math6 plays a role early in germ layer specification.

To verify early developmental expression of Math6, we utilized the GFP-Cre fusion protein to trace the fate of cells expressing Math6 in embryos derived from crosses between *Math6^+/EGFP-Cre^* and *Rosa26-LoxP-Stop-LoxP-LacZ* (R26R). Mice were harvested at E10.5, E14.5 and E18.5 and stained for β-galactosidase activity, which results from Cre-recombinase mediated excision of the stop cassette upstream of the ubiquitously expressed Rosa26 locus. β-Galactosidase expression was observed in all tissues at all stages examined (data not shown). Specifically within the pancreas all three lineages were marked: acinar, ductal and islet cells (Figure S3). These observations are in agreement with an early broad expression and role for Math6 in mouse embryonic development.

### *Math6* mRNA is specifically activated by bHLH factors

**Figure 8:**
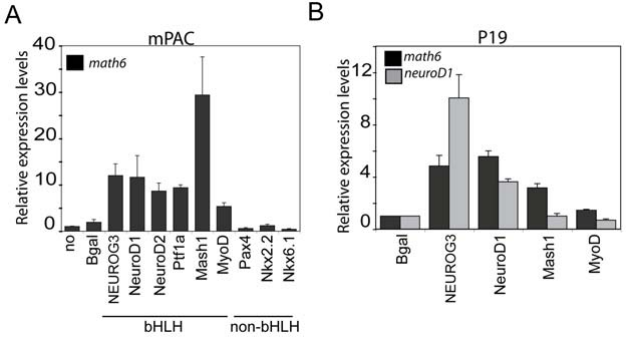
Regulation of *Math6* gene expression by multiple class B bHLH transcription factors. mPAC (A) or P19 (B) cells were transduced with adenoviruses expressing the indicated transcription factors or with a lentivirus expressing NeuroD2. Total RNA was isolated 48 h after viral treatment and *Math6* and endogenous *NeuroD1* mRNA levels were measured by real time PCR using GUS as internal standard. Values are expressed as fold-increase over basal (untreated cells). Bars represent mean±SEM of 3–5 independent experiments.

In order to gain further insight into the function of Math6 during development, we used the pancreas as a model to determine if specific gene families could activate its expression. In the mPAC duct cells system, adenovirally expressed NeuroD1, NeuroD2 and Ptf1a (p48) induced *Math6*, however Pax4, Nkx2-2 and Nkx6-1 did not (Figure 8A). Strikingly, like Neurog3, all the factors tested to date that activate Math6 belong to the bHLH family. Next we tested if non-pancreatic bHLH factors could also activate the *Math6* gene in the pancreatic duct cell system. Both neural Mash1 and myogenic MyoD increased *Math6* mRNA expression in mPAC cells, although with a marked variation in their degree of activation (Figure 8A).

To establish whether the observed regulation of the *Math6* gene by tissue-specific bHLH factors extended to other cell types, we studied Math6 activation in pluripotent mouse P19 embryonal carcinoma cells, which are capable of generating neurons after transient expression of neural bHLH factors [36]. We found that all tested bHLH factors induced *Math6* mRNA in p19 cells albeit to a lower degree (likely due to reduced transfection efficiency in P19 cells) than in mPAC cells (Figure 8A&B), indicating that activation of Math6 may be a common phenomenon in differentiation processes promoted by bHLH genes.

## Discussion

Transcription factors of the bHLH family are well-characterized regulators of differentiation processes in many tissues in vertebrates. In the embryonic pancreas, at least two *atonal*-related bHLH genes regulate the endocrine islet cell determination program: Neurog3 initiates endocrine differentiation and induces NeuroD1, which maintains endocrine differentiation [7] [16]. In this study, we demonstrate that another *atonal*-related bHLH protein, Math6, is transiently expressed *in vivo* within endocrine precursors. Math6 is positively regulated by Neurog3 and it can modulate the expression and proendocrine functions of Neurog3 in cell culture *in vitro*. In light of these data, we propose that this gene is a novel component of the Neurog3-dependent transcriptional cascade and it may play a role in endocrine cell genesis during pancreatic development.

The identification of the *Math6* gene as a target of Neurog3 in mouse duct cells prompted us to investigate the role of this little studied factor in islet cell differentiation. Using mice that carry the GFP reporter gene in one of the two *Math6* alleles, we found that Math6 appears in the pancreas around E12.5, and by E15.5, when Neurog3 expression peaks, nearly 50% of Neurog3+ cells co-express Math6. As development proceeds, pancreatic expression of both Math6 and Neurog3 decays and co-localization of these bHLH factors with islet hormones is not observed. The striking parallels in the expression patterns of Math6 and Neurog3 suggest tight regulatory interactions between them.

Despite the fact that Neurog3 induces *Math6*, it is clear from our studies that Math6 expression in the pancreas is not restricted to the endocrine compartment. Interestingly, in the E15.5 embryonic pancreas, Math6 is also found in peripheral epithelial cells known to express exocrine cell products at this developmental stage, suggesting that Math6 may participate in both endocrine and exocrine differentiation. In this vein, our observation that the pro-exocrine bHLH factor Ptf1a can induce Math6 expression *in vitro* (Figure 8) supports the notion that this bHLH factor may lie upstream of Math6 in the exocrine cell lineage [37], and suggests a general paradigm in which Math6 is induced broadly by class II bHLH factors that initiate differentiation in different cell lineages.

Generation of *Math6* null mice has demonstrated that, in addition to its probable roles in organ and tissue differentiation, Math6 is involved in early embryogenesis. However, due to early lethality of *Math6* null embryos, conditional gene knockout approaches will be necessary to address its roles in organogenesis. In an analogous manner, the Drosophila homologue of Math6, *net*, functions in at least two developmental stages in the fly. First, *net* is expressed in the ventral region of the blastoderm-stage embryo that is fated to become mesoderm in a pattern that overlaps the bHLH factor *twist*, suggesting that it may be playing a roles in mesoderm specification and myogenic pathways [30] [38]. Secondly, during postembryonic development, *net* regulates wing vein patterning and, by repressing EGF signalling, is instrumental in the determination of intervein *versus* vein cell fates in the wing [31]. In addition to its proposed roles during development, expression of Math6 in adult tissue is suggestive of additional functions of this factor in maintenance of differentiated phenotypes [32] [33]. In this regard, Hath6, the human orthologue of Math6, was identified as a flow-responsive gene in endothelial cells [32]. Interestingly, Math6 is expressed in differentiated kidney podocytes which have been recently shown to be highly sensitive to fluid sheer stress [39]. In the adult pancreas, even though GFP expression reaches undetectable levels by immunohistochemical techniques, Math6 mRNA transcripts are still detected in isolated pancreatic islets and in total pancreas by PCR (Figure 1 and data not shown). These results indicate that some Math6 expression remains in the adult organ. Identity of the cells responsible for this low level Math6 expression remains to be determined.

As a complementary strategy to mouse genetic models, we undertook an *in vitro* gain-of-function approach using mPAC cells (Figures 4 & 5) and demonstrated that Math6 alone cannot promote an endocrine gene expression program. It can, however, modulate the Neurog3-stimulated induction of some key genes. Ectopic Math6 expression decreased the Neurog3 stimulated induction of the *somatostatin and NeuroD1* genes. In contrast, other Neurog3-regulated genes such as *Pax4* or *Nkx2-2* were not affected by changes in Math6 expression. Collectively, these findings suggest that Math6 displays a degree of target specificity and may not globally modulate Neurog3 activity, although its ability to extinguish Neurog3 expression may eventually terminate all Neurog3 activity *in vivo*. Even though the precise mechanism underlying the functional interaction between Math6 and Neurog3 has not been addressed in the present paper, the physical association we observed between these two factors raises the possibility that Math6 is recruited to at least some Neurog3 target loci. Like other atonal homologues, Math6 contains a 12 amino acid-basic region that is known to be essential for Atonal-specific binding to DNA [29]. A future challenge will be to identify Math6 gene targets as well as to investigate the recruitment of Math6 to Neurog3 target promoters.

During pancreas development, the regulation of Neurog3 expression can be tightly controlled by a number of mechanisms. Autoregulation of the Neurog3 gene has been described as an important mechanism by which Neurog3 can efficiently control its own expression [9] [15]. This ability may not be exclusive to Neurog3, as autoregulatory loops have also been described for the proneural bHLH genes achaete, scute and atonal during *Drosophila* sensory organ development [40], NeuroD genes during neuronal differentiation [36] and myoD during myogenesis [41]. In this regard, our results reveal that changes in Math6 mRNA levels affect the ability of Neurog3 to activate its own gene; as overexpression of Math6 leads to an almost complete blockade of this positive autoregulatory loop, while it does not affect *Neurog3* gene activation by other regulators such as Mash1 (L.Sanchez, R.Gasa unpublished observations). Thus, it appears that levels of Math6 expression are tightly linked to the capacity for Neurog3 autoregulation in mPAC cells. Based on these findings, we speculate that high expression of Math6 may be involved in repression of the *Neurog3* gene: increased Math6 expression (concomitant with increases in Neurog3) may contribute to Neurog3 gene silencing. Furthermore, the coincidence of Math6 and Neurog3 expression in the pancreas further supports this idea. Using reporter gene strategies, it has been demonstrated that Neurog3 represses its own promoter both directly by displacing an activator, and indirectly by inducing a repressor [15]. Math6 with its intrinsic transcriptional repressor activity (Figure 6) appears to fulfil the role of a Neurog3-induced repressor of Neurog3 expression. However, despite the attractiveness of this hypothesis, future studies will further explore the mechanistic basis of the regulation of Neurog3 expression by Math6 to establish whether Math6 is a *bona fide* regulator of Neurog3 *in vivo.*

Lastly, this study provides the basis for further studies to investigate the role of Math6 in other bHLH-dependent differentiation processes. Here we have demonstrated that the induction of the Math6 gene is not specific to Neurog3. However, other pancreatic, neuronal and even myogenic bHLH factors also increase *Math6* mRNA levels upon their forced expression in duct cells. Furthermore, induction of Math6 by pancreatic and neuronal bHLH proteins is not a duct-cell specific phenomenon, as similar effects were observed in P19 cells. Likewise, the *math6* gene has been recently identified as a potential target of Neurog3 in embryonic stem cells [42] and it has been predicted to be a neurogenin/neuroD direct transcriptional target during neurogenesis using computational genomic-wide prediction analysis [43]. Based on our observations of a widespread expression of Math6 in the embryo together with the fact that bHLH proteins participate in multiple cell specification and differentiation events, it is tempting to speculate that activation of Math6 could represent a common downstream event elicited by multiple class B bHLH factors and thereby, be relevant to their functions in different developing tissues. In many respects, molecular regulation of endocrine differentiation parallels that of neurogenesis and even myogenesis, with a cascade of bHLH factors regulating cell fate determination, cell cycle withdrawal and induction of cell subtype-specific gene expression [44] [45] [46] [47]. Additionally, this negative regulation of Neurog3 may be of importance in regulating cell proliferation or apoptosis as it was recently demonstrated that Neurog3 overexpression in β-cells leads to increased apoptosis [48]. Moreover, common mechanisms like the recruitment of chromatin remodelling complexes to their target loci are used by both proneural and promyogenic bHLH factors to activate their target genes and induce differentiation [49] [50]. The implication of Math6 in other differentiation processes deserves further evaluation.

In summary, we have identified Math6 as a novel factor in the pancreatic developmental program. In addition, we suggest a widespread role for Math6 in the modulation of differentiation programs in various cell types during embryogenesis. Future studies including conditional gene knockout approaches will focus on further characterization of the role of Math6 in organism and endocrine pancreas differentiation. Hopefully, a greater understanding of Math6 function will enable further optimization of the development of cell-based therapies for both diabetes mellitus as well as other degenerative diseases.

## Materials and Methods

### RNA isolation and RT-PCR analysis

Total RNA was isolated from cell lines or mouse tissues using the RNeasy kit (Qiagen). First-strand cDNA was prepared using 2 µg of total RNA, the Superscript III RT kit and random hexamer primers (Invitrogen) in a total volume of 20 µl according to the manufacturer's instructions. 1/40 to 1/200 of the resulting cDNA was used as a template for conventional or real-time PCR reactions. All RNA samples were tested in the absence of reverse transcriptase. Real time PCR was performed on an ABI Prism 7900 sequence detection system using SybrGreen reagents (Applied Biosystems). Primer sequences are provided in Methods S1.

### Generation of Math6 knockout/knock-in mice

Gene targeting of the Math6 allele was carried out using a modified bacterial artificial chromosome [51] [52]. Briefly, a GFP-Cre fusion protein and an FRT-Neo cassette were inserted into the Math6 protein coding region within a BAC (RP22-157F13) derived from the 129S6/SvEvTac background containing aproximate 20 kb and 105 kb upstream and downstream of the Math6 gene respectively. The targeted BAC was purified using CsCl density gradient centrifugation, dialyzed, linearized and transfected into 129 (E14) mouse embryonic stem cells using electroporation. Stable clones were selected with 100 µg/ml G418 and screened for loss of the Math6 allele and gain of 1 copy of GFP using real-time PCR. Correctly targeted colonies were verified for only a single integration event using fluorescence in situ hybridation with the purified, labelled BAC DNA. Correctly targeted ES cells were injected into C57BL/6 blastocysts, chimeric mice were generated and backcrossed onto the C57BL/6 background. Following germline transmission of the targeted allele, mice were crossed to the Actin FLPe mice (Jackson Laboratories, Bar Harbor ME; B6.Cg-Tg (ACTFLPe) 9205Dym/J) for excision of the FRT-Neo cassette. Mice were genotyped using PCR with a three primer system (Methods S1) and standard reaction conditions and an annealing temperature of 66°C.

### Immunohistochemical and Immunocytochemical Analyses

Immunofluorescence assays were performed on cryosections of mouse tissues. Tissue was fixed overnight in 4% paraformaldehyde at 4°C. Following fixation, tissues were washed extensively in PBS, and then passed through 20% and 30% sucrose in PBS for 24 hrs each. Tissues were then embedded and frozen in Tissue-Tek (OCT Compound, Sakura). Tissues were sectioned at 10 µm, washed in PBS, permeabilized with 0.1% Triton X-100, blocked with 5% Goat serum (Invitrogen), 1% BSA (Sigma) and incubated with primary antibodies overnight at 4°C: rabbit anti-GFP (1∶1000; MBL), guinea pig anti-Neurog3(1∶1000;[3]), guinea pig anti-PDX1 (1∶2000; [3]), guinea pig anti-glucagon(1∶2000; Linco), guinea pig anti-insulin(1∶2000;Linco), rat anti-somatostatin(1∶200; Chemicon), hamster anti-mucin1(1∶200; NeoMarkers). Tissues were washed and incubated for 1 hr at room temperature with secondary antibodies: FITC conjugated goat anti-rabbit and Cy3 conjugated goat anti-guinea pig, goat anti-rat and goat anti-hamster (1∶200; 1∶400; Jackson Immunoresearch).

For localization of adenovirally-expressed Math6, mPAC cells were grown on chamber slides, treated with recombinant adenoviruses and, 48 h later, fixed in 4% paraformaldehyde (PFA) for 15min. Slides were sequentially incubated with monoclonal anti-myc antibody (Upstate) at 1∶200 dilution for 2 h, and cye2 coupled anti-mouse (Jackson ImmunoResearch) at 1∶1000 dilution for 1 h.

### Lineage Tracing Analyses

Math6^+/eGFPCre^ mice and the Rosa26-LoxP-Stop-Lox mice (Jackson Laboratories) were crossed, with midnight of the day in which a vaginal plug was observed being embryonic day 0. Embryos were harvested and pancreas and gut was dissected out and fixed briefly with 4% PFA. Tissues were then washed in PBS, permeabilized with 0.02% NP-40, 0.01% deoxycholate and then stained with X-Gal staining solution (2 mM MgCl2, 5 mM potassium ferricyanide, 5 mM potassium ferrocyanide, 20 mM Tris, pH 7.4 and 1 mg/ml X-Gal) overnight at 4°C. Once adequate staining had developed, tissues were washed with PBS, refixed with 4% PFA, dehydrated through ethanol to xylene and paraffin embedded. Paraffin blocks were sectioned at 5 µm thickness and stained as previously described [3].

### Expression and reporter vectors

The cDNAs for Math6 and Neurog3 were amplified by PCR from mouse E15.5 brain and pancreas respectively, using oligos Math6-5′, Math6-3′, Neurog3-5′ and Neurog3-3′ (Methods S1). A c-myc tag was added to the N-terminus of Math6 cDNA, and FLAG tags were added to the N-terminus of Neurog3 and NeuroD1 [15] cDNAs by PCR, and then cloned into the expression vector pCMV.TNT (Promega). The plasmid encoding hamster Nkx2-2 was previously used [35]. The one-hybrid expression vector encoding GAL4DBD-Math6 was generated by PCR using mouse Math6 cDNA as template, followed by in-frame ligation into the pM vector (Clontech). Plasmids expressing the fusion proteins GAL4-p300 (nt1737–2414) and GAL4-RbLP were kindly provided by Dr. Giordino (SHRO, PA) and Dr. Postigo (IDIBAPS; Barcelona, Spain).

A 1.8 Kb fragment of the Math6 gene upstream of the translation initiation site was amplified by PCR from mouse liver genomic DNA (oligos provided in Methods S1) and cloned upstream of the luciferase gene in the pFOXluc1 vector [35]. The luciferase reporter construct used in one-hybrid analysis has been described elsewhere [15].

### Cell culture and viral treatment

mPAC L20 cells were cultured in DMEM supplemented with 10% fetal bovine serum and antibiotics as previously described [9]. P19 cells (ATCC) were maintained in alpha-MEM with 7.5% calf serum, 2.5% fetal bovine serum, 2 mM L-glutamine and antibiotics. For viral treatment, 250,000 mPAC or 100,000 P19 cells were seeded onto 6-well plates the day before infection. Adenoviruses were added at a multiplicity of infection (moi) of 40 and incubated for 2 h (mPAC) or 5 h (P19) at 37°C in culture medium. Then, cells were cultured for 48 h, unless otherwise indicated.

An adenovirus expressing Math6 with a c-myc N-terminal tag was constructed by homologous recombination in HEK293 cells as previously described [53]. The adenovirus expressing Ptf1a was a kind gift from Dr. A. Skoudy [54] (IMIM; Barcelona, Spain). All other adenoviruses and the lentivirus for NeuroD2 were described elsewhere [9] [34].

### Luciferase reporter assays

For Math6 promoter assays, mPAC cells were seeded onto 24-well plates 24 h before treatment. Plasmid DNA (1 ug Firefly reporter+20 ng Renilla vector (pGL4.74, Promega) was mixed with 7 equivalents of linear 22 KDa polyethylenimine (PEI, ExGen 500, Fermentas) in serum free medium and then combined with 10^7^ pfu of the indicated recombinant adenoviruses. The DNA/ExGen/adenovirus mixture was then added to the cells and incubated at 37°C for 4 h. Cells were harvested 24 h later and assayed for luciferase activity.

For one-hybrid assays, 60,000 mPAC cells were plated onto 24-well plates 24 h before transfection. 500 ng of the firefly luciferase reporter construct were cotransfected with 5 ng of pGL4.74 vector and increasing amounts (20 to 100 ng) of the vectors encoding GAL4DBD fusion proteins. Metafectene reagent (Biontex) was used for all transfections under conditions recommended by the manufacturer. 48 h after transfection, cells were collected and luciferase activities assayed using the Dual Luciferase Kit from Promega. Reporter assays were performed in duplicate and data corresponds to at least 4 independent transfection experiments.

### Chromatin immunoprecipitation

mPAC cells were fixed with 1% formaldehyde and lysed in SDS buffer (EDTA 10 mM, Tris.HCl 50 mM pH 8.1, SDS1%). Chromatin was sheared to 1 Kb using sonication and then cleared by centrifugation. Immunoprecipitations were carried out overnight at 4°C using 400 µg protein and a rabbit anti-serum raised against a GST-human Neurog3 (amino acids 1–95) protein or normal rabbit serum or no antibody as controls. Protein A/G PLUS-agarose (Santa Cruz) blocked with salmon sperm DNA (0.2 mg/ml) was used to immunoprecipitate the complexes. Immunoprecipitates were washed as previously described [55] and subjected to PCR analysis with primers outlined in Methods S1.

### Coimmunoprecipitation and western blotting

mPAC cells were seeded onto 6 cm plates 24 h before treatment. Adeno-assisted transfection was carried out mixing 4 ug of expression vector, 7 equivalents of PEI and 10^7^ pfu of Ad.CMV.math6 and incubating this mixture with the cells for 16 h at 37°C. Cells were harvested 24 h later, washed 2 times with PBS and lysed in coIP buffer (NaCl 100 mM, Tris-HCl pH 7.5 20 mM, EDTA 1 mM, Igepal CA-630 1%, NaF 5 mM, 10% protease inhibitor cocktail from SIGMA). Lysates were incubated for 15 min at 4°C and cellular debris was removed by centrifugation at 10,000× g for 10 min at 4°C. Cellular lysates (500 ug) were then immunoprecipitated with anti-FLAG (SIGMA), anti-Nkx2-2 (Hybridoma Bank) or mouse IgG1 (Sigma) overnight at 4°C. Immunoconjugates were recovered using proteinG-coupled dynabeads (Invitrogen) and, after 3 washes with coIP buffer, they were eluted by boiling in SDS-Laemli buffer. To prepare whole cell lysates, mPAC cells were lysed in triple detergent lysis buffer (TrisHCl pH 8 50 mM, NaCl 150 mM, SDS 0.1%, Igepal CA-6301%, Na deoxycholate 0.5%).

Proteins from both whole cell extracts and immunoprecipitates were separated by PAGE-SDS electrophoresis, transferred to PVDF membranes (Perkin Elmer) and incubated overnight at 4°C with rabbit anti-human Neurog3 (same antibody that was used for ChIP assays) at 1∶2000 dilution, mouse anti-myc (Upstate) at 1∶1000 dilution, mouse anti-FLAG (Sigma) at 1∶1000 dilution and mouse anti-Nkx2-2 (Hybridoma bank) at 1∶2000 dilution. Blots were visualized with ECL Reagent (Pierce Biotechnology).

## Supporting Information

Figure S1 Generation and Screening of the Math6-GFPCre knockin mouse. The first step (A1) in generation of the targeting allele was recombination of the GFPCre-pA-FRT-SV40Neo-FRT targeting cassette into the BAC (RP22-157F13) replacing the first two exons of Math6. Clones were picked for their dual resistance to chloramphenicol and kanamycin and screened using both PCR, pulse-field gel electrophoresis and sequencing for correct recombination. DNA was then isolated using CsCl density gradient purification, linearized with PI-SceI and electroporated into 129 (E14) mouse embryonic stem cells (2). Stable clones were selected with 100 µg/ml G418 and screened for presence of BAC-vector backbone sequence using the following primers that flank the PI-SceI site: FCL104; 5′-GGA AGG AGC TGA CTG GGT TG-3′, FCL105; 5′-TGA GTC GTA TTA GCG GCC G-3′, FCL106; 5′-AGG AGG AGC GAC TCA AGC C-3′, FCL107; 5′-CGT GAT AGC CGT TGT ATT CAG C-3′ using standard PCR. Those clones that did not contain vector sequences were further screened for loss of the Math6 allele and gain of 1 copy of GFP using real-time PCR (B–E). This was accomplished by designing primer and probe sets that amplify: beta-actin (B; FCL111; 5′-TTC AAC ACC CCA GCC ATG TA-3′, FCL112; 5′-TGT GGT ACG ACC AGA GGC ATA C-3′, FCL113; 5′/56FAM/TAG CCA TCC AGG CTG TGC TGT CCC/3IAbFQ/-3′), the 5′ region of the targeted Math6 gene downstream of the homology arm (C; FCL113; 5′-CAA GCG GAA AGG CAA GGA′-3, FCL114; 5′-TCC AAG TCC AAT CGG AAA GTT T-3′, FCL109; 5′-/5TET/CCA TTC GCG CGC CGC A/3BHQ_1/-3′), the 3′ region of the targeted Math6 gene upstream of the homology arm (D; FCL115; 5′-TGG GCA GAA GCT CTC CAA A-3′, FCL116; 5′-CGT GCC AGG GAC AAG ATG TA-3′, FCL110; 5′/56-TAMN/TTA CAG GCA ATC CTC AGG ATG GCC A/3BHQ_2/-3′) and GFP (E; FCL150; 5′-AGT CCG CCC TGA GCA AAG A-3′, FCL151; 5′-GGC GGT CAC GAA CTC CAG-3′, FCL149; 5′-/5TET/CCC AAC GAG AAG CGC GAT CAC A/3BHQ_1/-3′). Note the increase in 1 cycle in clone A1 compared to the other clones for both the 5′ and 3′ arms (C&D). Positive clones were then used for fluorescence in situ hybridization (FISH) analyses with labeled BAC DNA and with labeled GFP sequences (F). Wild-type and targeted clones (A1) contained largely the same BAC sequence and two chromosomal spots were observed in all cells. This ruled out the inclusion of an extra copy of the BAC at a non-homologously-recombined locus (cf left panel vs middle panel). GFP FISH further confirmed the presence of only one chromosomal locus containing the GFP transgene (right panel). Correct clones (eg A1) were injected into C57BL/6 blastocysts and germline transmission was achieved. Mice were then crossed with the Actin FLPE mice (Jackson; Tg(ACTFLPe)9205Dym; (Rodriguez et al., Nat Genet, 25, 139–140, 2000) and then intercrossed to obtain knockout blastocysts (G) that did not express eGFP at this time.
(2.60 MB TIF)

Figure S2 Neurog3 induces hath6 mRNA in human duct cells. PANC-1 and HPDE-E6E7 cells were treated with adenoviruses encoding Neurog3 (AdCMV-NEUROG3) or B-galactosidase (AdCMV-Bgal) at a moi of 50 for 2 h. Then, virus-containing media was replaced and cells were cultured for an additional 48 h-period. Total RNA was isolated and gene expression for Neurog3, Hath6 and beta-actin genes was assessed by RT-PCR. Oligos used were as follows: 5′-hath6 (5′-CAT CAG CGC AGC CTT CGA G); 3′-hath6 (5′- AGG CGA TCC TCA GGA TGG CC); 5′-Neurog3 (5′-GGG TCC CTC TAC TCC CCA GTC TCC); Neurog3 (5′- CTC AAG CAG GCG GAA AAG GTG G); 5′-actin (5′- TGA GAG GGA AAT CGT GCG TG) and 3′-actin (5′- TGC TTG CTG ATC CAC ATC TGC)
(0.37 MB TIF)

Figure S3 Math6 lineage in the pancreas from the Math6+/EGFP-Cre mice. Heterozygous Math6+/EGFP-Cre mice were crossed with the Rosa26-Lox-Stop-Lox mice (Jackson; Soriano, Nat Genet 21, 70–71, 1999) and harvested at E14.5 (A–D) or E18.5 (E–H). Pancreas and attached gut was removed and stained with X-Gal. Tissues were then dehydrated, paraffin embedded and sectioned into 5 µm thin sections and peroxidase stained for Pdx1 (A&B), neurogenin3 (C&D), insulin (E), glucagon (F), somatostatin (G) or amylase (H) as described in Methods. Math6 promoter activity (as denoted by Cre-mediated excision) was present in all cell types and tissues within the embryo: indicating early, widespread activity. Scale bars = 50 µm.
(9.86 MB TIF)

Methods S1 (0.07 MB DOC)

## References

[1] Guillemot F (2005). Cellular and molecular control of neurogenesis in the mammalian telencephalon.. Curr Opin Cell Biol.

[2] Jensen J, Heller RS, Funder-Nielsen T, Pedersen EE, Lindsell C (2000). Independent development of pancreatic alpha- and beta-cells from neurogenin3-expressing precursors: a role for the notch pathway in repression of premature differentiation.. Diabetes.

[3] Schwitzgebel VM, Scheel DW, Conners JR, Kalamaras J, Lee JE (2000). Expression of neurogenin3 reveals an islet cell precursor population in the pancreas.. Development.

[4] Sommer L, Ma Q, Anderson DJ (1996). neurogenins, a novel family of atonal-related bHLH transcription factors, are putative mammalian neuronal determination genes that reveal progenitor cell heterogeneity in the developing CNS and PNS.. Mol Cell Neurosci.

[5] Tajbakhsh S, Buckingham M (2000). The birth of muscle progenitor cells in the mouse: spatiotemporal considerations.. Curr Top Dev Biol.

[6] Zammit PS, Carvajal JJ, Golding JP, Morgan JE, Summerbell D (2004). Myf5 expression in satellite cells and spindles in adult muscle is controlled by separate genetic elements.. Dev Biol.

[7] Gradwohl G, Dierich A, LeMeur M, Guillemot F (2000). neurogenin3 is required for the development of the four endocrine cell lineages of the pancreas.. Proc Natl Acad Sci U S A.

[8] Apelqvist A, Li H, Sommer L, Beatus P, Anderson DJ (1999). Notch signalling controls pancreatic cell differentiation.. Nature.

[9] Gasa R, Mrejen C, Leachman N, Otten M, Barnes M (2004). Proendocrine genes coordinate the pancreatic islet differentiation program in vitro.. Proc Natl Acad Sci U S A.

[10] Grapin-Botton A, Majithia AR, Melton DA (2001). Key events of pancreas formation are triggered in gut endoderm by ectopic expression of pancreatic regulatory genes.. Genes Dev.

[11] Heremans Y, Van De Casteele M, in't Veld P, Gradwohl G, Serup P (2002). Recapitulation of embryonic neuroendocrine differentiation in adult human pancreatic duct cells expressing neurogenin 3.. J Cell Biol.

[12] Johansson KA, Dursun U, Jordan N, Gu G, Beermann F (2007). Temporal control of neurogenin3 activity in pancreas progenitors reveals competence windows for the generation of different endocrine cell types.. Dev Cell.

[13] Jensen J, Pedersen EE, Galante P, Hald J, Heller RS (2000). Control of endodermal endocrine development by Hes-1.. Nat Genet.

[14] Lee JC, Smith SB, Watada H, Lin J, Scheel D (2001). Regulation of the pancreatic pro-endocrine gene neurogenin3.. Diabetes.

[15] Smith SB, Watada H, German MS (2004). Neurogenin3 activates the islet differentiation program while repressing its own expression.. Mol Endocrinol.

[16] Naya FJ, Huang HP, Qiu Y, Mutoh H, DeMayo FJ (1997). Diabetes, defective pancreatic morphogenesis, and abnormal enteroendocrine differentiation in BETA2/neuroD-deficient mice.. Genes Dev.

[17] Sosa-Pineda B, Chowdhury K, Torres M, Oliver G, Gruss P (1997). The Pax4 gene is essential for differentiation of insulin-producing beta cells in the mammalian pancreas.. Nature.

[18] Sussel L, Kalamaras J, Hartigan-O'Connor DJ, Meneses JJ, Pedersen RA (1998). Mice lacking the homeodomain transcription factor Nkx2.2 have diabetes due to arrested differentiation of pancreatic beta cells.. Development.

[19] Sander M, Sussel L, Conners J, Scheel D, Kalamaras J (2000). Homeobox gene Nkx6.1 lies downstream of Nkx2.2 in the major pathway of beta-cell formation in the pancreas.. Development.

[20] Collombat P, Mansouri A, Hecksher-Sorensen J, Serup P, Krull J (2003). Opposing actions of Arx and Pax4 in endocrine pancreas development.. Genes Dev.

[21] Gierl MS, Karoulias N, Wende H, Strehle M, Birchmeier C (2006). The zinc-finger factor Insm1 (IA-1) is essential for the development of pancreatic beta cells and intestinal endocrine cells.. Genes Dev.

[22] Huang HP, Liu M, El-Hodiri HM, Chu K, Jamrich M (2000). Regulation of the pancreatic islet-specific gene BETA2 (neuroD) by neurogenin 3.. Mol Cell Biol.

[23] Smith SB, Gasa R, Watada H, Wang J, Griffen SC (2003). Neurogenin3 and hepatic nuclear factor 1 cooperate in activating pancreatic expression of Pax4.. J Biol Chem.

[24] Watada H, Scheel DW, Leung J, German MS (2003). Distinct gene expression programs function in progenitor and mature islet cells.. J Biol Chem.

[25] Mellitzer G, Bonne S, Luco RF, Van De Casteele M, Lenne-Samuel N (2006). IA1 is NGN3-dependent and essential for differentiation of the endocrine pancreas.. Embo J.

[26] Gu G, Wells JM, Dombkowski D, Preffer F, Aronow B (2004). Global expression analysis of gene regulatory pathways during endocrine pancreatic development.. Development.

[27] Petri A, Ahnfelt-Ronne J, Frederiksen KS, Edwards DG, Madsen D (2006). The effect of neurogenin3 deficiency on pancreatic gene expression in embryonic mice.. J Mol Endocrinol.

[28] White P, May CL, Lamounier RN, Brestelli JE, Kaestner KH (2008). Defining pancreatic endocrine precursors and their descendants.. Diabetes.

[29] Inoue C, Bae SK, Takatsuka K, Inoue T, Bessho Y (2001). Math6, a bHLH gene expressed in the developing nervous system, regulates neuronal versus glial differentiation.. Genes Cells.

[30] Ledent V, Vervoort M (2001). The basic helix-loop-helix protein family: comparative genomics and phylogenetic analysis.. Genome Res.

[31] Brentrup D, Lerch H, Jackle H, Noll M (2000). Regulation of Drosophila wing vein patterning: net encodes a bHLH protein repressing rhomboid and is repressed by rhomboid-dependent Egfr signalling.. Development.

[32] Wasserman SM, Mehraban F, Komuves LG, Yang RB, Tomlinson JE (2002). Gene expression profile of human endothelial cells exposed to sustained fluid shear stress.. Physiol Genomics.

[33] Ross MD, Martinka S, Mukherjee A, Sedor JR, Vinson C (2006). Math6 expression during kidney development and altered expression in a mouse model of glomerulosclerosis.. Dev Dyn.

[34] Gasa R, Mrejen C, Lynn FC, Skewes-Cox P, Sanchez L (2008). Induction of pancreatic islet cell differentiation by the neurogenin-neuroD cascade.. Differentiation.

[35] Watada H, Mirmira RG, Leung J, German MS (2000). Transcriptional and translational regulation of beta-cell differentiation factor Nkx6.1.. J Biol Chem.

[36] Farah MH, Olson JM, Sucic HB, Hume RI, Tapscott SJ (2000). Generation of neurons by transient expression of neural bHLH proteins in mammalian cells.. Development.

[37] Rose SD, Swift GH, Peyton MJ, Hammer RE, MacDonald RJ (2001). The role of PTF1-P48 in pancreatic acinar gene expression.. J Biol Chem.

[38] Moore AW, Barbel S, Jan LY, Jan YN (2000). A genomewide survey of basic helix-loop-helix factors in Drosophila.. Proc Natl Acad Sci U S A.

[39] Friedrich C, Endlich N, Kriz W, Endlich K (2006). Podocytes are sensitive to fluid shear stress in vitro.. Am J Physiol Renal Physiol.

[40] zur Lage PI, Powell LM, Prentice DR, McLaughlin P, Jarman AP (2004). EGF receptor signaling triggers recruitment of Drosophila sense organ precursors by stimulating proneural gene autoregulation.. Dev Cell.

[41] Thayer MJ, Weintraub H (1990). Activation and repression of myogenesis in somatic cell hybrids: evidence for trans-negative regulation of MyoD in primary fibroblasts.. Cell.

[42] Serafimidis I, Rakatzi I, Episkopou V, Gouti M, Gavalas A (2008). Novel effectors of directed and Ngn3-mediated differentiation of mouse embryonic stem cells into endocrine pancreas progenitors.. Stem Cells.

[43] Seo S, Lim JW, Yellajoshyula D, Chang LW, Kroll KL (2007). Neurogenin and Neurod direct transcriptional targets and their regulatory enhancers.. EMBO J.

[44] Guillemot F (1999). Vertebrate bHLH genes and the determination of neuronal fates.. Exp Cell Res.

[45] Halevy O, Novitch BG, Spicer DB, Skapek SX, Rhee J (1995). Correlation of terminal cell cycle arrest of skeletal muscle with induction of p21 by MyoD.. Science.

[46] Liu Y, Encinas M, Comella JX, Aldea M, Gallego C (2004). Basic helix-loop-helix proteins bind to TrkB and p21(Cip1) promoters linking differentiation and cell cycle arrest in neuroblastoma cells.. Mol Cell Biol.

[47] Berkes CA, Tapscott SJ (2005). MyoD and the transcriptional control of myogenesis.. Semin Cell Dev Biol.

[48] Dror V, Nguyen V, Walia P, Kalynyak TB, Hill JA (2007). Notch signalling suppresses apoptosis in adult human and mouse pancreatic islet cells.. Diabetologia.

[49] de la Serna IL, Ohkawa Y, Berkes CA, Bergstrom DA, Dacwag CS (2005). MyoD targets chromatin remodeling complexes to the myogenin locus prior to forming a stable DNA-bound complex.. Mol Cell Biol.

[50] Seo S, Richardson GA, Kroll KL (2005). The SWI/SNF chromatin remodeling protein Brg1 is required for vertebrate neurogenesis and mediates transactivation of Ngn and NeuroD.. Development.

[51] Valenzuela DM, Murphy AJ, Frendewey D, Gale NW, Economides AN (2003). High-throughput engineering of the mouse genome coupled with high-resolution expression analysis.. Nat Biotechnol.

[52] Yang Y, Seed B (2003). Site-specific gene targeting in mouse embryonic stem cells with intact bacterial artificial chromosomes.. Nat Biotechnol.

[53] Becker TC, Noel RJ, Coats WS, Gomez-Foix AM (1994). Use of recombinant adenovirus for metabolic engineering of mammalian cells.. Methods Cell Biol.

[54] Rovira M, Jane-Valbuena J, Marchand M, Savatier P (2007). Viral-mediated coexpression of Pdx1 and p48 regulates exocrine pancreatic differentiation in mouse ES cells.. Cloning Stem Cells.

[55] Lynn FC, Smith SB, Wilson ME, Yang KY, Nekrep N (2007). Sox9 coordinates a transcriptional network in pancreatic progenitor cells.. Proc Natl Acad Sci U S A.

