# Supplementary figures and images for: Identification of the bHLH Factor Math6 as a Novel Component of the Embryonic Pancreas Transcriptional Network
Source: PLoS One. 2008 Jun 18;3(6):e2430. doi: 10.1371/journal.pone.0002430 (PMC2413403; doi:10.1371/journal.pone.0002430)

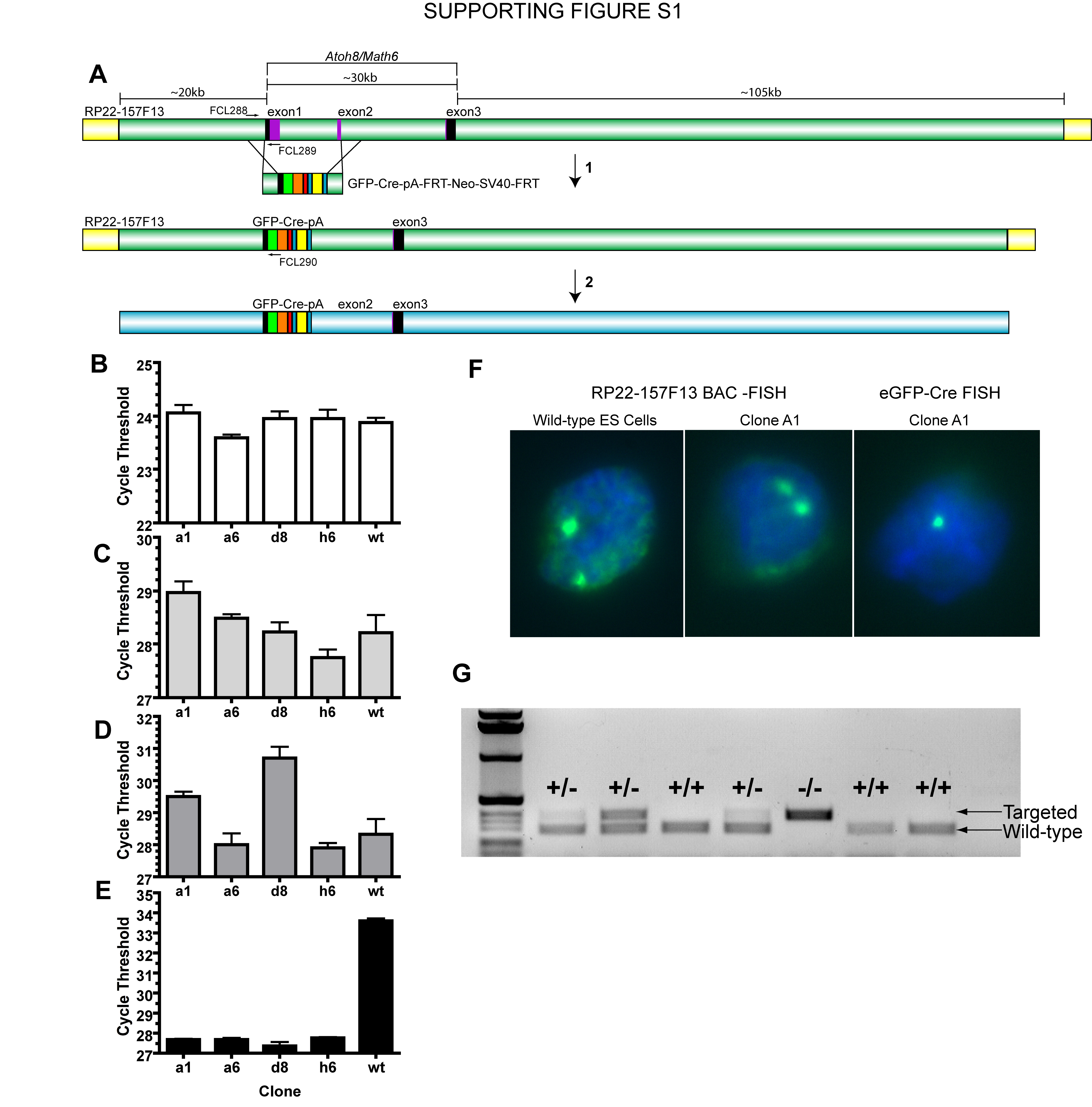

Supplement: Figure S1 — Generation and Screening of the Math6-GFPCre knockin mouse. The first step (A1) in generation of the targeting allele was recombination of the GFPCre-pA-FRT-SV40Neo-FRT targeting cassette into the BAC (RP22-157F13) replacing the first two exons of Math6. Clones were picked for their dual resistance to chloramphenicol and kanamycin and screened using both PCR, pulse-field gel electrophoresis and sequencing for correct recombination. DNA was then isolated using CsCl density gradient purification, linearized with PI-SceI and electroporated into 129 (E14) mouse embryonic stem cells (2). Stable clones were selected with 100 µg/ml G418 and screened for presence of BAC-vector backbone sequence using the following primers that flank the PI-SceI site: FCL104; 5′-GGA AGG AGC TGA CTG GGT TG-3′, FCL105; 5′-TGA GTC GTA TTA GCG GCC G-3′, FCL106; 5′-AGG AGG AGC GAC TCA AGC C-3′, FCL107; 5′-CGT GAT AGC CGT TGT ATT CAG C-3′ using standard PCR. Those clones that did not contain vector sequences were further screened for loss of the Math6 allele and gain of 1 copy of GFP using real-time PCR (B–E). This was accomplished by designing primer and probe sets that amplify: beta-actin (B; FCL111; 5′-TTC AAC ACC CCA GCC ATG TA-3′, FCL112; 5′-TGT GGT ACG ACC AGA GGC ATA C-3′, FCL113; 5′/56FAM/TAG CCA TCC AGG CTG TGC TGT CCC/3IAbFQ/-3′), the 5′ region of the targeted Math6 gene downstream of the homology arm (C; FCL113; 5′-CAA GCG GAA AGG CAA GGA′-3, FCL114; 5′-TCC AAG TCC AAT CGG AAA GTT T-3′, FCL109; 5′-/5TET/CCA TTC GCG CGC CGC A/3BHQ_1/-3′), the 3′ region of the targeted Math6 gene upstream of the homology arm (D; FCL115; 5′-TGG GCA GAA GCT CTC CAA A-3′, FCL116; 5′-CGT GCC AGG GAC AAG ATG TA-3′, FCL110; 5′/56-TAMN/TTA CAG GCA ATC CTC AGG ATG GCC A/3BHQ_2/-3′) and GFP (E; FCL150; 5′-AGT CCG CCC TGA GCA AAG A-3′, FCL151; 5′-GGC GGT CAC GAA CTC CAG-3′, FCL149; 5′-/5TET/CCC AAC GAG AAG CGC GAT CAC A/3BHQ_1/-3′). Note the increase in 1 cycle in clone A1 compared to the other clones for both the [file pone.0002430.s001.tif]

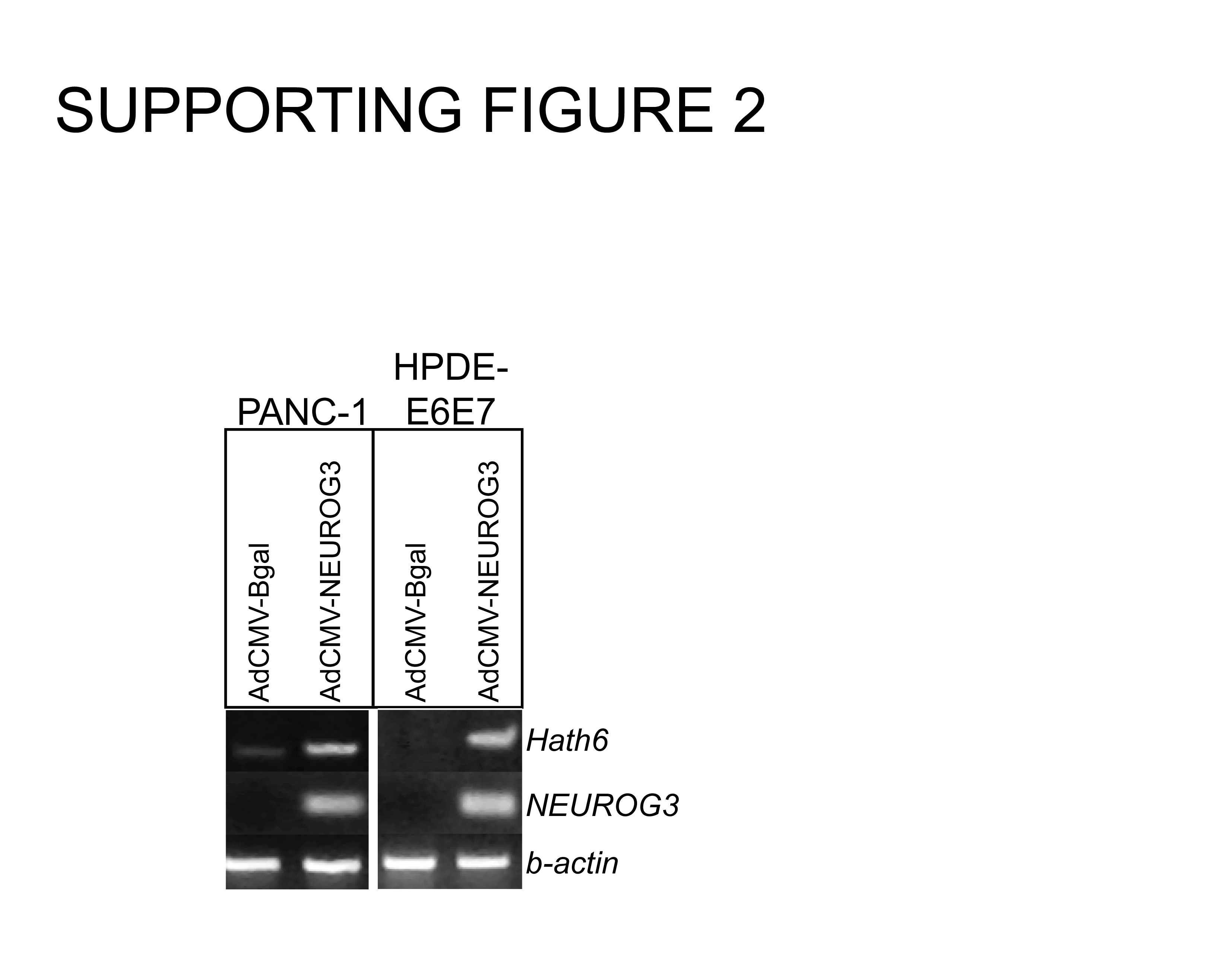

Supplement: Figure S2 — Neurog3 induces hath6 mRNA in human duct cells. PANC-1 and HPDE-E6E7 cells were treated with adenoviruses encoding Neurog3 (AdCMV-NEUROG3) or B-galactosidase (AdCMV-Bgal) at a moi of 50 for 2 h. Then, virus-containing media was replaced and cells were cultured for an additional 48 h-period. Total RNA was isolated and gene expression for Neurog3, Hath6 and beta-actin genes was assessed by RT-PCR. Oligos used were as follows: 5′-hath6 (5′-CAT CAG CGC AGC CTT CGA G); 3′-hath6 (5′- AGG CGA TCC TCA GGA TGG CC); 5′-Neurog3 (5′-GGG TCC CTC TAC TCC CCA GTC TCC); Neurog3 (5′- CTC AAG CAG GCG GAA AAG GTG G); 5′-actin (5′- TGA GAG GGA AAT CGT GCG TG) and 3′-actin (5′- TGC TTG CTG ATC CAC ATC TGC) (0.37 MB TIF) [file pone.0002430.s002.tif]

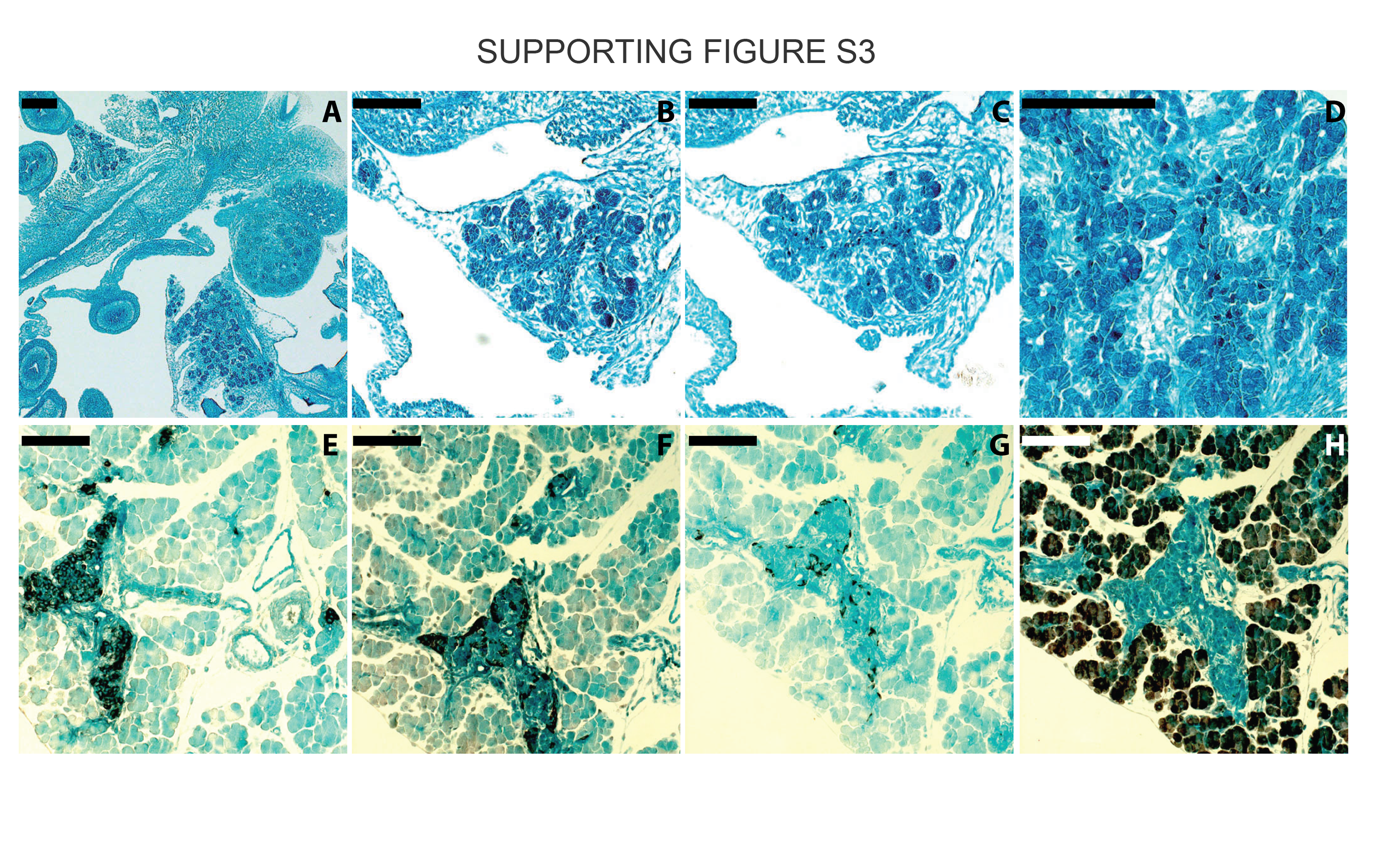

Supplement: Figure S3 — Math6 lineage in the pancreas from the Math6+/EGFP-Cre mice. Heterozygous Math6+/EGFP-Cre mice were crossed with the Rosa26-Lox-Stop-Lox mice (Jackson; Soriano, Nat Genet 21, 70–71, 1999) and harvested at E14.5 (A–D) or E18.5 (E–H). Pancreas and attached gut was removed and stained with X-Gal. Tissues were then dehydrated, paraffin embedded and sectioned into 5 µm thin sections and peroxidase stained for Pdx1 (A&B), neurogenin3 (C&D), insulin (E), glucagon (F), somatostatin (G) or amylase (H) as described in Methods. Math6 promoter activity (as denoted by Cre-mediated excision) was present in all cell types and tissues within the embryo: indicating early, widespread activity. Scale bars = 50 µm. (9.86 MB TIF) [file pone.0002430.s003.tif]
