# Supplementary material for: Identification of the bHLH Factor Math6 as a Novel Component of the Embryonic Pancreas Transcriptional Network
Source: PLoS One. 2008 Jun 18;3(6):e2430. doi: 10.1371/journal.pone.0002430 (PMC2413403; doi:10.1371/journal.pone.0002430)
Supplement: Methods S1 — (0.07 MB DOC) [file pone.0002430.s004.doc]

**PRIMER SEQUENCES**

**Genotyping:**

FCL288 (5’-TGC AGA AAT GCT GGC AGG G), FCL289 (5’-CGG ACA CGC TGA ACT TGT GG), FCL290 (5’-GAT GTG CTT CAT GGC GAG GAC)

**cDNA Expression constructs:**

Math6-5’ (5’-CCA TCT AGA ATG AAG CAC ATC CCG GTC CTC) and Math6-3’(5’-GAC AAG CTT TCA CTC CTT CCG TTT CTT GGC)

Neurog3-5’ (5’-ATA CCA TGG GAG AAC TAG GAT GGC G) and Neurog3-3’ (5’-ACG TCT AGA GTC TCT TCA CAA GAA GTC TG)

**Amplification Math6 Promoter:**

5’-GGT ACC TGA CCA GTC CTC ACG GGT TGT CCT T and 5’-GGA TCC GGC GAG GAC GGC GTG AGG CTG GG

**Chromatin Immunoprecipitation:**

| PROMOTER | FORWARD | **REVERSE** |
| --- | --- | --- |
| Math6 | TGA GTGATCTTGCGAACTGGG | **GATGTGCTTCATGGCGAGGA** |
| Pax4 | CCAACGATCCAGGCTCTACA | **CACCGGGTTTGGGGCTAAT** |
| **Beta actin** | **AACAAGAGGCCACACAAATAGG** | **CAGATGTACAGGAATAGCCTCCG** |

**Real time PCR and conventional RT-PCR**:

| GENE | FORWARD | REVERSE | **User for** |
| --- | --- | --- | --- |
| Beta actin | TGAGAGGGAAATCGTGCGTG | TGCTTGCTGATCCACATCTGC | **RT-PCR** |
| Glucagon | ACCATTTACTTTGTGGCTGGATTG | TCCGCAGAGATGTTGTGAAGATG | **RT-PCR** |
| Gus | CTCATCTGGAATTTCGCCGA | GGCGAGTGAAGATCCCCTTC | **REALTIME** |
| Hes-1 | TGCTACCCCAGCCAGTGTCAAC | TTCTTGCCCTTCGCCTCTTC | **RT-PCR** |
| Hes-6 | GCTTCGCTGCTGGCTACATC | ACCTGGGGGACTGGACAATG | **RT-PCR** |
| IAPP | CTCCAAACTGCCAGCTGTCC | TCCGTTTGTCCATCTGAGGG | **REALTIME** |
| Insulin | AGCGTGGCTTCTTCTACACACC | CCAGCTCCAGTTGTGCCACT | **RT-PCR** |
| Islet-1 | CGGACAGACATGATGGTGGTT | GCAAGGCGAAGTCACTCAGTACT | **RT-PCR** |
| Math6 | GCATCTCAGAGCTTGGCACC | AATCCTCAGGATGGCCAGTTT | **RT-PCR** |
| Math6 | AGCCAAGAAACGGAAGGAGTG | TCTGGCAGCATCTTGAGGAAG | **REALTIME** |
| NeuroD1 | CACGCAGAAGGCAAGTGTC | TCTTTTTGGGACCCCGTCTC | **RT-PCR** |
| NeuroD1 | CACGCAGAAGGCAAGTGTC | CGCTCTCGCTGTATGATTTGG | **REALTIME** |
| Neurogenin3 (hm) | GGGTCCCTCTACTCCCCAGTCTCC | CTCAAGCAGGCGGAAAAGGTGG | **RT-PCR/ REALTIME** |
| Neurogenin3 | CGCACCATGGCGCCTCATCCCTTGG | CAGAGGATCCTCTTCACAAGAAGTCTGAG | **RT-PCR** |
| Neurogenin3 | TTCTCATCGGTACCCTTGCTG | GCAGACTCACCAGGAAGTATGG | **REALTIME** |
| Nkx2.2 | GCTGACCAACACAAAGACG | CTGGGTCTCCTTGTCATTG | **RT-PCR** |
| Nkx2.2 (1a) | GAACAGCAAGCTAGCCGAGG | CGATCAGTCCATATAAGGCTGG | **REALTIME** |
| Nkx6.1 | TGGACAGCAAATCTTCGCCCTG | TGTTGTAATCGTCGTCATCCTC | **RT-PCR** |
| Pax4 | GTGTTGGCTCCAGTTCTTCC | AACCAAACCCTCACCGTGTC | **RT-PCR** |
| Pax4 | GAGTACCCTGCTCTTTTTGCC | ACTCGATTGATAGAGGACACACT | **REALTIME** |
| Pdx-1 | ACATCTCCCCATACGAAGTGCC | AAGTTCAACATCACTGCCAGCTCC | **RT-PCR** |
| Somatostatin | AGGCAAGGAAGATGCTGTCCTG | CAATTTCTAATGCAGGGTCAAGTTG | **RT-PCR** |
| Somatostatin | ACCCCAGACTCCGTCAGTTTC | ATCATTCTCTGTCTGGTTGGGC | **REALTIME** |
| **TBP** | **ACCCTTCACCAATGACTCCTATG** | **ATGATGACTGCAGCAAATCGC** | **RT-PCR** |
